# The Iowa Gambling Task and the three fallacies of dopamine in gambling disorder

**DOI:** 10.3389/fpsyg.2013.00709

**Published:** 2013-10-08

**Authors:** Jakob Linnet

**Affiliations:** ^1^Research Clinic on Gambling Disorders, Aarhus University HospitalAarhus, Denmark; ^2^Clinical Department, Center of Functionally Integrative Neuroscience, Medical School of Aarhus UniversityAarhus, Denmark; ^3^Division on Addiction, Cambridge Health AllianceCambridge, MA, USA; ^4^Department of Psychiatry, Harvard Medical School, Harvard UniversityCambridge, MA, USA

**Keywords:** gambling disorder, Iowa Gambling Task (IGT), dopamine, addiction, positron-emission tomography

## Abstract

Gambling disorder sufferers prefer immediately larger rewards despite long term losses on the Iowa Gambling Task (IGT), and these impairments are associated with dopamine dysfunctions. Dopamine is a neurotransmitter linked with temporal and structural dysfunctions in substance use disorder, which has supported the idea of impaired decision-making and dopamine dysfunctions in gambling disorder. However, evidence from substance use disorders cannot be directly transferred to gambling disorder. This article focuses on three hypotheses of dopamine dysfunctions in gambling disorder, which appear to be “fallacies,” i.e., have not been supported in a series of positron emission tomography (PET) studies. The first “fallacy” suggests that gambling disorder sufferers have lower dopamine receptor availability, as seen in substance use disorders. However, no evidence supported this hypothesis. The second “fallacy” suggests that maladaptive decision-making in gambling disorder is associated with higher dopamine release during gambling. No evidence supported the hypothesis, and the literature on substance use disorders offers limited support for this hypothesis. The third “fallacy” suggests that maladaptive decision-making in gambling disorder is associated with higher dopamine release during winning. The evidence did not support this hypothesis either. Instead, dopaminergic coding of reward prediction and uncertainty might better account for dopamine dysfunctions in gambling disorder. Studies of reward prediction and reward uncertainty show a sustained dopamine response toward stimuli with maximum uncertainty, which may explain the continued dopamine release and gambling despite losses in gambling disorder. The findings from the studies presented here are consistent with the notion of dopaminergic dysfunctions of reward prediction and reward uncertainty signals in gambling disorder.

## Introduction

Impaired performance on the Iowa Gambling Task (IGT) is associated with a range of substance use disorders and behavioral addictions including gambling disorder. The term “gambling disorder” was recently introduced in version 5 of the Diagnostic Statistical Manual (DSM) (American Psychiatric Association DSM 5, 2013) as a separate chapter on “behavioral addiction” under the substance use classification. In DSM-IV (American Psychiatric Association DSM-IV, 1994) the disorder was classified as “pathological gambling” under “impulse control disorders.” The change in classification and grouping has two important implications. First it suggests that gambling disorder shares the clinical characteristics of substance use disorders rather than impulse control disorders. This change is significant because it underscores the relevance of comparing gambling disorder with other forms of addiction with regard to for instance clinical epidemiological and neurobiological aspects of the disorder. Second it uniquely differentiates gambling disorder as a “behavioral addiction” from other substance use disorders which emphasizes that addiction can be purely behavioral and need not involve the intake of exogenous substances.

The research approach on neurobiological markers of IGT performance in gambling disorder presented here focuses on these two distinctions. On the one hand it identifies common features of dopaminergic dysfunctions and impaired IGT performance in gambling disorder and related substance use disorders; on the other hand it seeks to identify unique patterns of dopamine dysfunctions in relation to impaired IGT performance of gambling disorder sufferers compared with evidence from the literature on substance use disorders.

The present article suggests that there are three hypotheses of dopaminergic dysfunctions in gambling disorder, which appear to be fallacies, i.e., it has not been possible to find support for the hypotheses in a series of positron emission tomography (PET) studies on gambling disorder. The first hypothesis suggests that gambling disorder sufferers have lower baseline binding potentials, as seen in substance use disorders; the second hypothesis suggests that gambling activity is associated with higher dopamine release in gambling disorder, i.e., that gambling disorder sufferers have dopaminergic hypersensitivity toward gambling; the third hypothesis suggests that winning is associated with higher dopamine release in gambling disorder, i.e., that gambling disorder sufferers have dopaminergic hypersensitivity toward winning. Finally, it is suggested that reward prediction and reward uncertainty signals, which are learning mechanisms associated with dopamine release, might better account for the dopaminergic dysfunctions and impaired IGT performance in gambling disorder, and evidence is presented to support this vantage point.

### The Iowa gambling task in substance use disorders and gambling disorder

The IGT is an executive functions task, which simulates real life decision making in the way that it factors reward and punishment (Bechara et al., 1994). Individuals choose between four decks of cards labeled A, B, C, and D, with the objective to win as much money as possible. In decks A and B (“disadvantageous decks”), choosing a card is followed by an immediately high gain of money, but at unpredictable trials the selection is followed by a high loss, leading to a net loss over time. In decks C and D (“advantageous decks”), the immediate gain is smaller, but the future loss is also smaller, leading to a net gain over time. Other versions of the IGT have been developed, where, for instance, the contingencies are reversed (Bechara et al., 2002).

Originally, findings on the IGT showed that patients suffering from lesions in the ventromedial prefrontal cortex (sometimes referred to as the orbitofrontal cortex) have a higher preference for immediate rewards despite negative future consequences (Bechara et al., 1994, 2000). These findings led to the suggestion that lesion patients suffer from insensitivity to future consequences. The findings of impaired decision-making in lesion patients were replicated in individuals suffering from substance use disorders, suggesting that these individuals prefer immediate rewards despite negative long-term consequences (Bechara et al., 2001; Bechara, 2003). The impairments were linked to prefrontal cortex dysfunctions, based on the evidence from lesion patients. The findings were later extended to gambling disorder, where gambling disorder sufferers show decision-making impairments similar to individuals suffering from substance use disorders (Grant et al., 2000; Petry, 2001; Cavedini et al., 2002; Goudriaan et al., 2005, 2006a; Linnet et al., 2006).

Linnet et al. (2006) investigated “chasing one's losses,” a key diagnostic symptom of gambling disorder. The authors compared 61 gambling disorder sufferers with 39 healthy controls. Gambling disorder sufferers were recruited through a treatment center, and healthy controls were selected from a pool of first-year psychology students. All participants completed a modified version of the IGT called the “Mouse Game” where individuals had to help a mouse gather cheese, rather than winning money. The contingencies were the same as the IGT, but units were converted into grams of cheese and the winning and losing sounds were removed, in order to reduce the association with gambling. The decks on the Mouse Game were stacked with 100 cards, such that participants could not deplete the decks during trials; the last 40 cards were added to the original 60-card stack on the IGT.

The study aimed at developing a quantifiable behavioral measure of chasing in a gambling situation where decision-making and skill would determine the outcome of the game. It was hypothesized that gambling disorder sufferers would have impaired IGT performance and increased *episodic chasing* (i.e., sequences of persistent poor choices leading to losses) compared with healthy controls, suggesting that they would be less likely to use negative feedback to change their behavior. To define chasing on the IGT, an index of behavior focused on choice sequences was compiled. *Advantageous choice sequence* was defined as five consecutive advantageous decisions (cards from deck C or D) and a *disadvantageous choice sequence* as five consecutive disadvantageous decisions (cards from deck A or B). The chance of choosing five consecutive good or bad cards at random is 2^−5^ = 0.03125 (*p* < 0.05).

The result showed that gambling disorder sufferers had significantly higher chasing on the IGT than healthy controls (*df* = 4, *F* = 3.61, *p* ≤ 0.01). The advantageous and disadvantageous chasing episodes were distributed evenly throughout the game. In other words, individuals did not solely have, e.g., advantageous decision-making sequences in the beginning of the game and disadvantageous sequences toward the end of the game. Rather, a pattern emerged for players with several behavior episodes in which both advantageous and disadvantageous decisions were present at the beginning of the game, developing into a “learning curve” of predominantly advantageous or disadvantageous sequences as the game unfolded. These results are consistent with the notion that gambling disorder sufferers are more impulsive and less likely to adopt a long term advantageous strategy, even in the face of negative feedback, than healthy controls. They are also consistent with the notion of reduced PFC functions and/or dopamine dysfunctions in the disorder.

### Substance use disorders and the dopamine system

Using drugs such as cocaine, amphetamine, and methamphetamine increases extracellular dopamine in the synaptic cleft, and binds more dopamine to the dopamine receptors of the synapses (Stahl, 2006). In healthy individuals increased dopamine binding to dopamine D_2/3_ receptors is associated with a higher self-reported hedonic pleasure (Volkow et al., 2002a). The hedonic pleasure from drug liking is linked to two factors: (1) the baseline level of dopamine receptor availability before drug use; and (2) the change in dopamine receptor availability following drug use. Dopamine receptor availability is measured using, for instance, PET, where a radioactive ligand such as [^11^C]raclopride is injected into the blood stream, and measured based on its binding properties. Raclopride binds to available dopamine D_2/3_ receptors in the brain, and the raclopride binding potential is an index of dopamine receptor availability:

(1) $${\text{BP}}_{\text{ND}}=\frac{B_{max}-B}{V_{d}K_{d}}$$

where *Bmax* is the maximum binding capacity of the receptors, *B* is the binding of the radioligand, *V*_*d*_ is the volume distribution, and *K*_*d*_ is the ligand's half-saturation concentration.

A higher baseline raclopride binding potential is interpreted as a higher number of dopamine receptors available for binding; a higher (positive) change in raclopride binding potential from a baseline to an experimental condition is interpreted as an increased release of dopamine because more dopamine is bound to the receptors in the experimental condition. Substance use disorders are associated with lower baseline dopamine receptor availability and reduced dopamine release from drug use.

#### Baseline levels of dopamine receptor availability

Healthy individuals with lower baseline dopamine receptor availability have higher hedonic pleasure from drug use than individuals with higher levels of dopamine receptor availability (Volkow et al., 1999, 2002b). These findings have been interpreted to suggest that lower baseline dopamine receptor availability is a risk factor for developing substance use disorders, while higher receptor availability could help prevent developing substance use disorder.

In a study of 15 methamphetamine use disorder sufferers and 20 healthy control subjects Volkow et al. (2001) found that the methamphetamine use disorder sufferers had significantly lower dopamine binding than control subjects. The authors note that the results could either reflect a pre-conditioned vulnerability toward addiction, or a down-regulation of dopamine receptors or loss of dopamine transporters following the methamphetamine use disorder.

Later, Volkow et al. (2006) compared dopamine receptor availability of non-addicted family members from families with a history of alcoholism and family members from families without a history of alcoholism. Individuals from families with a history of alcoholism had significantly lower baseline dopamine receptor availability than individuals from families without alcoholism. The results are consistent with the notion that higher baseline dopamine receptor availability is a protective factor against alcoholism and substance dependence. Individuals from families without a history of alcoholism may have been “protected” from developing substance use disorder by higher dopamine receptor availability, while individuals from families with a history of alcoholism may be at risk for developing substance use disorder due to lower receptor availability.

#### Dopamine release and substance use

Healthy volunteers show a significant correlation between change in dopamine binding and hedonic response to substance use; individuals with larger dopamine release from substance use report larger hedonic response (Volkow et al., 2002a). However, the evidence of change in dopamine release and hedonic response in substance use disorders is more complex (Volkow et al., 1997, 2002a, 2008; Kalivas and Volkow, 2005). Volkow et al. (1997) investigated dopamine release and hedonic response from methamphetamine use in 20 detoxified cocaine use disorder individuals and 23 healthy controls. Participants were given a moderate dosage of intravenously injected methamphetamine, a substance similar to cocaine. The results confirmed previous reports that cocaine use disorder individuals had lower baseline dopamine receptor availability than healthy controls. They further showed that healthy controls had significantly increased dopamine release throughout the striatum and felt significantly more “high” and “restlessness” from drug use compared to cocaine use disorder individuals.

The results suggest a blunted dopaminergic effect toward methamphetamine and reduced feelings of “high” in cocaine use disorder sufferers. In other words, individuals with cocaine use disorder neither have increased dopamine release nor increased pleasure from using drugs similar to cocaine compared with healthy control individuals. Substance use disorders therefore cannot be explained by increased dopamine release from substance use or higher pleasure from dopamine release per se. The involvement of dopamine in substance use disorders is more complex.

### Gambling disorder and the dopamine system

The dopamine system is sensitive to behavioral stimulation related to monetary reward (Koepp et al., 1998; Breiter et al., 2001; Zald et al., 2004). For instance, Koepp et al. (1998) found that skilled video game players had significant dopamine release in the striatum when playing a video game for money.

Another line of evidence of the role of dopamine in gambling disorder comes from the literature on gambling disorder in Parkinson's disease sufferers in agonist treatment. Parkinson's disease sufferers, who are treated with dopamine agonists, have significantly higher prevalence of gambling disorder than individuals who receive other forms of treatment (Grosset et al., 2006; Lu et al., 2006; Weintraub et al., 2006). Agonist treatment is also associated with other impulse control disturbances such as hypersexuality, compulsive shopping, and compulsive eating (Steeves et al., 2009). These data suggest that certain changes to the dopamine system is associated with increased risk of addiction and impulse control disorders, including gambling disorder. While the dopaminergic mechanism behind the increased risk of gambling disorder is currently unknown, Steeves et al. (2009) found significant dopamine release in the ventral striatum of Parkinson's disease patients suffering from gambling disorder who gambled for money. Furthermore, de la Fuente-Fernandez et al. (2002) found significant dopamine release in the ventral striatum of Parkinson's patients expecting a drug reward in placebo trials. The authors concluded that the dopamine release was mediated by the expectation of reward. Unlike gambling disorder sufferers, Parkinson's disease sufferers in agonist treatment with gambling disorder have reduced binding potentials as a consequence of Parkinson's disease, and they therefore represent an atypical case of gambling disorder. For this reason the present review predominantly focuses on dopaminergic dysfunctions in gambling disorder without Parkinson's disease.

While use of substances such as cocaine is associated with dopamine release throughout the striatum, the ventral striatum is specifically involved in drug expectation and monitoring of reward (Delgado et al., 2000; de la Fuente-Fernandez et al., 2002), and this region appears to be central to gambling disorder and substance use disorder (Reuter et al., 2005; Abler et al., 2006; Linnet et al., 2010; 2011a,b). Evidence from the animal literature also supports the involvement of the ventral striatum in drug seeking and addictive behavior (Dalley et al., 2007; Uhl, 2007; Doya, 2008). Dopaminergic dysfunctions in the ventral striatum might therefore contribute to the decision making impairments on the IGT seen in gambling disorder. However, while substance use disorder and gambling disorder may share a common neurobiological basis, there might be differences in dopaminergic dysfunctions related to drug use and gambling.

The present review examines similarities and differences in dopaminergic dysfunctions between substance use disorder and gambling disorder based on a series of articles investigating the relation between dopaminergic neurotransmission and IGT performance in gambling disorder (Linnet et al., 2010, 2011a,b, 2012). In the study we scanned gambling disorder sufferers and healthy controls with PET using the radioligand [^11^C]raclopride to measure dopaminergic neurotransmission during a baseline and a gambling condition of the IGT. In the baseline condition participants played a non-decision IGT similar to that of Bolla et al. (2003, 2004), where the computer automatically instructed the participants which cards to choose, and no winning or losing sounds were used; during the gambling scan participants chose freely between the decks, and received auditory feedback when winning or losing. Since each PET scan lasted 60 min, and it only takes ~20 min to administer the IGT, three versions of the IGT were used: the regular ABCD version, and subsequent KLMN and QRST versions, where the contingencies between decks become increasingly ambiguous. Raclopride binding potentials (BP_ND_) and change in binding potential (ΔBP_ND_) between baseline and gambling condition were recorded. Higher raclopride binding potentials (BP_ND_) indicate a higher number of D_2/3_ receptors available for dopamine binding. Decreased raclopride binding potentials from baseline to gambling condition indicate dopamine release because dopamine occupies more receptors during gambling and leaves fewer receptors available for raclopride binding. Raclopride binding potentials were measured using the ERLiBiRD method (Gjedde et al., 2005), and a ventral striatum mask using criteria similar to those of Mawlawi et al. (2001) was used to determine the anatomical location of the ventral striatum. Other masks were used for the putamen and caudate nucleus. The raclopride emission recordings were co-registered with structural MR images for each individual using MNI tools, and transformed into a common stereotaxic coordinate space (Talairach and Tournoux, 1988).

The study findings gave rise to the notion of the three “fallacies” of the role of dopamine in gambling disorders. Specifically, we found no support for the hypotheses that: (1) gambling disorder sufferers have lower baseline dopamine receptor availability; (2) gambling disorder sufferers have increased dopamine release when gambling; and (3) gambling disorder sufferers have increased dopamine release when winning.

#### Fallacy 1: gambling disorder sufferers have lower baseline dopamine receptor availability

While studies of substance use disorder have consistently and independently shown lower baseline dopamine receptor availability throughout the brain in substance use disorder (Volkow et al., 1997, 2001), we found no such differences in gambling disorder (Linnet et al., 2010, 2011a,b, 2012). Linnet et al. (2010) compared raclopride binding potentials (BP_ND_) in the ventral striatum of 16 gambling disorder sufferers and 15 healthy controls. The results showed no significant differences in baseline binding potentials between the two groups. Follow-up studies expanding the cohort to 18 gambling disorder sufferers and 16 healthy controls (Linnet et al., 2012) confirmed these findings throughout the striatum. Later independent studies support that gambling disorder sufferers do not differ in baseline dopamine receptor availability compared with healthy controls (Clark et al., 2012; Boileau et al., 2013).

The findings differ from the literature on substance use disorder, where individuals with substance use disorder have significantly lower binding potentials than healthy controls (Volkow et al., 2001). The differences in results may suggest a down-regulation of receptor availability as a consequence of substance use disorder, which is not present in gambling disorder. Co-morbidity between gambling disorder and substance use disorders is generally high (Crockford and el-Guebaly, 1998; Ibanez et al., 2001; Kausch, 2003; Petry et al., 2005; Dannon et al., 2006; Kessler et al., 2008), and presence of substance use disorders increases severity of gambling disorder (Rush et al., 2008) or risk thereof (el-Guebaly et al., 2006). However, our population of gambling disorder sufferers (Linnet et al., 2010, 2011a,b, 2012) was screened for substance use disorders. It is therefore, possible that lower levels of baseline dopamine binding potentials are found in individuals suffering from co-morbidity of gambling disorder and substance use disorders. More importantly, the findings might have implications for understanding the role of dopamine in the behavioral addictions (Holden, 2001; Shaffer and Kidman, 2003; Petry, 2006; Potenza, 2006; Grant et al., 2010), and may indicate neurobiological distinctions between behavioral addictions and substance use disorders at the level of the striatum and ventral striatum.

#### Fallacy 2: gambling disorder sufferers have increased dopamine release when gambling

**Figure 1:**
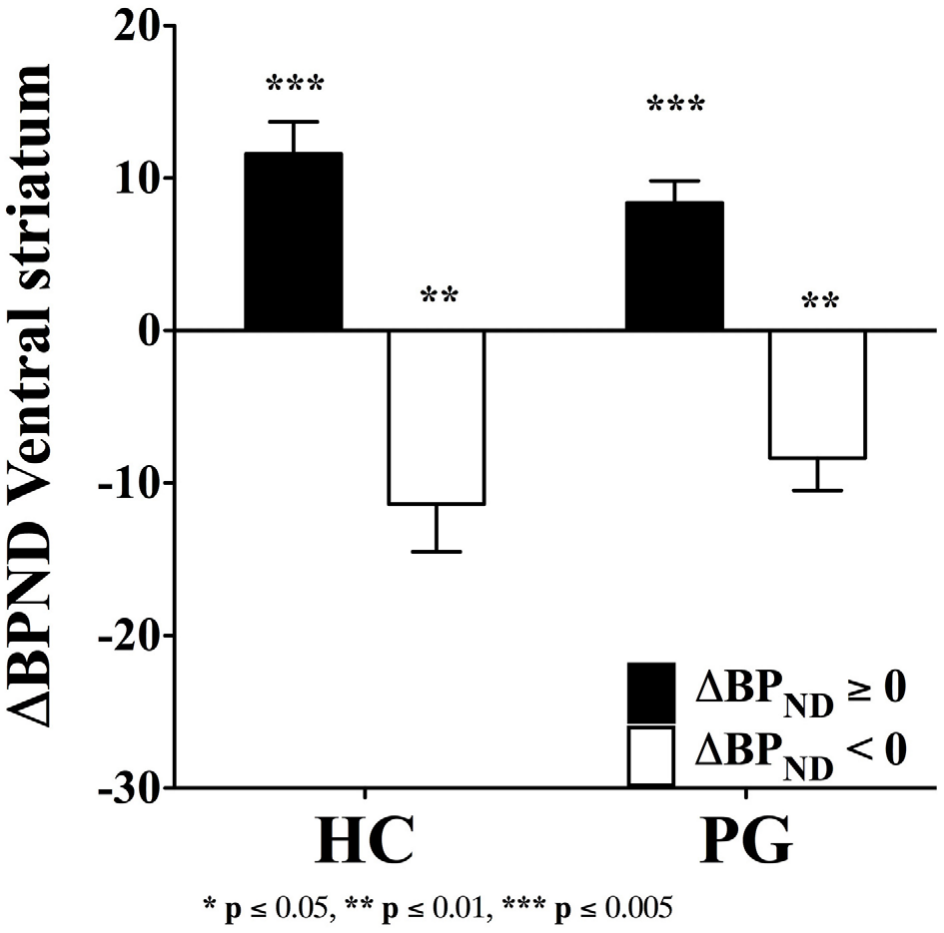
**Binding potential changes (ΔBP_ND_) in ventral striatum.** Gambling disorder sufferers (PG, *n* = 8) and healthy controls (HC, *n* = 5) show no significant differences in magnitude of dopamine release from baseline to gambling condition (ΔBP_ND_ = 0, black bars). Similarly, gambling disorder sufferers (PG, *n* = 8) and healthy controls (HC, *n* = 9) show no significant differences in magnitude of dopamine inhibition from baseline to gambling condition (ΔBP_ND_ < 0, white bars). The ordinate shows the change in binding potential (ΔBP_ND_), while the error bars indicate Standard Error Means (SEM). Star symbols (*) indicate *p*-values in comparison to baseline. Reprint with permission from Linnet et al. (2011b).

Despite the evidence of a blunted dopamine response in substance use disorder (Volkow et al., 1997), the fallacy of a hyperdopaminergic response to reward in substance use disorder has transcended into the field of gambling disorder. The dopamine system is sensitive to behavioral stimulation related to monetary reward (Koepp et al., 1998; Breiter et al., 2001; Zald et al., 2004), which has lead to the suggestion of dopamine dysfunctions in gambling disorder (Holden, 2001). However, the evidence of a hyperdopaminergic response to reward in gambling disorder is mixed. Steeves et al. (2009) reported an increased dopamine response to winning in a PET study of Parkinson's disease patients with gambling disorder compared with Parkinson's disease patients without gambling disorder. However, we (Linnet et al., 2011b) found that some gambling disorder sufferers and healthy controls had significant dopamine release in the ventral striatum when gambling on the IGT, compared with the no-gambling condition, but we did not find differences between the two groups in the magnitude of dopamine release (see Figure 1). Figure 1 shows gambling disorder sufferers (PG) and healthy controls (HC) with positive changes in binding potential (BP_ND_ ≥ 0, black bars) from baseline to gambling condition, suggesting dopamine release. It can be seen from the figure that the two groups do not differ in the magnitude of dopamine release from gambling. Similarly, we found no group differences in negative changes in binding potential (BP_ND_ < 0, white bars), suggesting dopamine inhibition. Comparing gambling disorder sufferers and healthy controls throughout the striatum revealed similar results (Linnet et al., 2012).

Even if the evidence supported the fallacy of a hyperdopaminergic response to reward in substance use disorder, PET activation paradigms used to study substance use disorder and gambling disorder may be too different to enable conclusions about differences or similarities in dopamine release toward reward in the two populations, because administering a drug may activate the dopamine system in a very different way than a gambling simulation.

More importantly, the blunted dopamine response to reward in substance use disorder might poorly explain the mechanisms of addiction and a possible common neurobiological pathway of addiction. What then, might explain dopaminergic dysfunctions in addiction? Robinson and Berridge (1993, 2000, 2003, 2008) have suggested that dopaminergic response to *anticipated* reward (“wanting”), rather than the reward itself (“liking”) constitutes a fundamental dopaminergic mechanism in addiction. In addiction “wanting” increases, while “liking” decreases, and this decrease in “liking” might correspond with the blunted dopamine response to reward. Dysfunctions in dopaminergic response to *anticipated* reward, on the other hand, might constitute a common mechanism of addiction, because it occurs in the absence of reward, and therefore may have similar (dys)function, whether the reward is food, drugs or gambling. This mechanism might correspond to the common clinical symptoms in addictions such as preoccupation or craving. It might also be involved in continued use despite negative consequences such as depressed mood or loss chasing.

In gambling disorder dopaminergic coding of uncertainty might represent a dysfunctional reward anticipation, which reinforces the gambling behavior despite losses (see the section on “Dopaminergic coding of reward prediction and uncertainty in gambling”).

#### Fallacy 3: gambling disorder sufferers have increased dopamine release when winning

Steeves et al. (2009) found that Parkinson's disease sufferers with gambling disorder had increased dopamine release when winning on a modified version of the IGT compared with Parkinson's disease sufferers without gambling disorder. The task was rigged with a 3:1 reward vs. penalty ratio, so it always produced an overall gain. The authors attributed the increased dopamine release in gambling disorder to the gains from gambling, and suggested that the increase might reflect a priming effect or premorbid dopaminergic hypersensitivity of the ventral striatal circuits.

**Figure 2:**
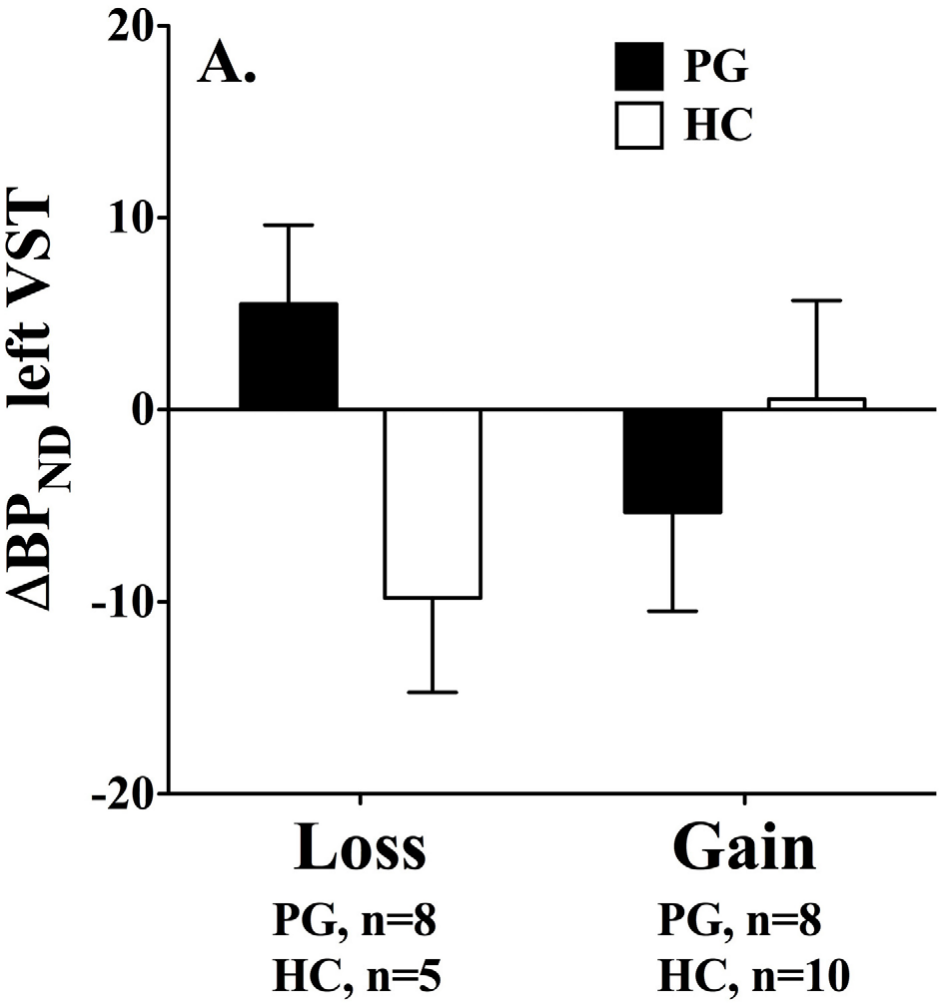
**Binding potential changes (ΔBP_ND_) in left ventral striatum.** Gambling disorder sufferers who lose money (PG, black bar, *n* = 8) have significantly higher dopamine release in the left ventral striatum than healthy controls (HC, white bar, *n* = 5). Gambling disorder sufferers who win money (PG, black bar, *n* = 8) do not differ in dopamine release from healthy controls (HC, white bar, *n* = 10). Mean and Standard Errors are illustrated in the bars and error bars, respectively. Dopamine release results in positive values because raclopride binding potentials decrease from baseline to gambling condition (baseline > gambling = positive value). Conversely, dopamine inhibition results in negative values because raclopride binding potentials increase from baseline to gambling condition (baseline < gambling = negative value). Reprint with permission from Linnet et al. (2010).

However, the results, are in contrast to findings by (Linnet et al. 2010). We found that gambling disorder individuals who lost money had significantly higher dopamine release in the left ventral striatum than healthy controls, *F*_(1, 29)_ = 5.52, *p* < 0.05 (*p* < 0.02 one-tailed). Furthermore, a Two-Way ANOVA showed a significant interaction effect, *F*_(2, 28)_ = 4.18, *p* = 0.05, where dopamine release was associated with losses in the gambling disorder group and gains in the healthy control group, see Figure 2. No group differences were found in the right ventral striatum.

These apparent differences raise the question of whether or not alternative models can better explain the role of dopamine release in relation to gains and losses in gambling disorder. Dopaminergic coding of reward prediction and uncertainty might offer such a model.

### Dopaminergic coding of reward prediction and uncertainty in gambling

**Figure 3:**
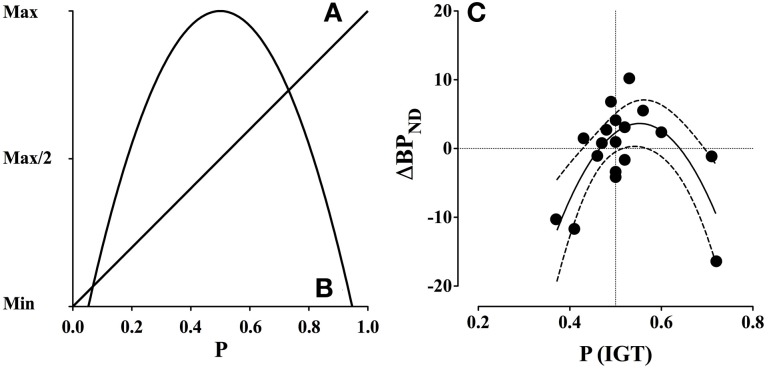
**Expected value and uncertainty as a function of reward probability and dopamine release (ΔBP_ND_). (A)** Expected value is a linear function of reward probability, where the expected value is minimal at *p* = 0.0 and maximal at *p* = 1.0. **(B)** Uncertainty, defined as variance, is a quadratic function of reward probability, where uncertainty is maximal at *p* = 0.5 and minimal at *p* = 0.0 and *p* = 1.0. **(C)** Gambling disorder sufferers (PG) show an inverted U-shaped function between binding potential (ΔBP_ND_) in the combined striatum and probability of selecting advantageous decks [P(IGT)]. The interaction is significant (*p* < 0.005) and accounts for 53.4% of the variation, *R*^2^(18) = 0.534. The dashed lines indicate confidence intervals (95% CE). Positive ΔBP_ND_ values suggest dopamine release, because dopamine occupies more receptors during the gambling condition, while negative ΔBP_ND_ values suggest that dopamine occupies fewer receptors. Reprint with permission from Linnet et al. (2012). **(A, B)** are amended from Figure 1, **(C)** is amended from Figure 2.

Reward prediction error in the dopamine system refers to a mechanism that updates positive and negative reward predictions of a stimulus. The mechanism constitutes a neural correlate of the mathematical and behavioral Rescorla-Wagner learning rule (Schultz, 2006). For instance, in random binary outcome situations (e.g., reward vs. no-reward) the expected value (EV) is the average value that can be expected from a given stimulus, which is a linear function of reward probability (Figure 3A). In contrast, uncertainty, defined as the variance (σ^2^) of the probability distribution is the mean squared deviation from the expected value, which is an inverted quadratic function of reward probability (Schultz et al., 2008) (Figure 3B).

Midbrain and striatal dopamine coding of expected value and uncertainty follow linear and quadratic functions similar to their mathematical expressions (Fiorillo et al., 2003; Preuschoff et al., 2006; Schultz, 2006). Fiorillo et al. (2003) found distinct phasic and sustained midbrain activation toward reward probability in monkeys using electrophysiological measures of dopamine neurons in the ventral midbrain areas A8, A9, and A10. Phasic dopamine activation was larger in anticipation of stimuli with larger reward probability, and smaller in anticipation of stimuli with smaller reward probability. The sustained activation was largest toward stimuli with maximum uncertainty (*P* = 0.5) and declined toward higher and lower probabilities. The phasic and sustained activation patterns were distinct both in terms of timing of signal and dopamine neurons coding the response.

Preuschoff et al. (2006) found distinct neural coding of expected value and uncertainty in the ventral striatum of healthy men and women in a monetary card-guessing task. Expected value was linearly associated with early anticipatory blood oxygen level dependent (BOLD) activation, such that higher reward probabilities were associated with higher anticipatory BOLD activation, and lower reward probabilities were associated with lower anticipatory BOLD activation. In contrast, uncertainty showed an inverse quadratic association with late anticipatory BOLD activation, such that the highest BOLD activation was seen around maximum uncertainty (*P* = 0.5) and the lowest BOLD activation was seen around maximum certainty (*P* = 1.0 and *P* = 0.0).

Linnet et al. (2012) hypothesized that dopaminergic coding of outcome uncertainty on the IGT in gambling disorder would follow the reward prediction error signal, i.e., have the properties of an inverted U-shaped curve. The IGT consists of two “advantageous” and two “disadvantageous” decks that will lead to long-term gains and losses, respectively. The person is free to chose between decks, and the IGT performance can therefore be expressed as the probability (*P*) of advantageous deck selection, such that the variance is (1 − *P*)* *P*.

The results confirmed the hypothesis of a significant inverse quadratic relationship between dopamine release and IGT performance among gambling disorder sufferers, which was strongest in the combined striatum, *F*_(2, 15)_ = 9.28, *p* = 0.002 (see Figure 3C). The quadratic relationship between dopamine release and IGT performance did not reach significance level in the healthy control group.

These results have implications for the findings by Steeves et al. (2009) and Linnet et al. (2010). In the study by Steeves et al. (2009) the computer program used a random sequence generator to determine the card sequence, and the outcome was therefore random, or uncertain, even though it always resulted in an overall gain. It is therefore possible that the dopaminergic coding in gambling disorder was also related to the variance of the task and not solely to the overall gain. The findings by Linnet et al. (2010) that gambling disorder sufferers had increased dopamine release during periods of losing—not winning—could suggest that dopamine release reinforces the gambling behavior despite losses, and preclude the individual from inhibiting the gambling behavior in order to stop gambling or avoid further losses. Both studies can be explained in terms of dopaminergic coding of reward prediction and uncertainty.

Since variance is a common feature in all forms of gambling, and since uncertainty and variance is maximized in most forms of gambling, the dopaminergic response to maximum variance might reinforce the gambling behavior despite losses, and this might constitute a common underlying mechanism in gambling disorder. The odds structure in the most addictive forms of gambling are optimized toward maximum uncertainty and variance, where the payback percentage is around 80–99% (e.g., slot machines typically have payback rates of 82–90%, and black jack has payback rates as high as 99%). Since the odds only slightly favor the house, and the variance is maximized, these games provide the optimal conditions for dopaminergic coding of uncertainty and reinforcement of gambling behavior despite losses, which may underlie clinical behavior such as “chasing one's losses.”

From the perspective of gambling disorder, the outcome of winning or losing does not matter in the short term. What matters is that the game properties will always lead to losses in the long run, and the variance in outcome will always lead to dopaminergic reinforcement of the gambling behavior. This combination constitutes an inherent risk for gambling disorder sufferers and individuals at risk for developing gambling disorder.

Dopaminergic coding of reward prediction and uncertainty offers a model for explaining why: (1) gambling disorder sufferers are drawn toward the risk and uncertainty of gambling; (2) gambling disorder sufferers continue gambling despite losses; and (3) gambling disorder sufferers do not adapt an advantageous strategy despite negative feedback. At the same time it is clear that this model does not account for all behavior. For instance, our data are limited to PET and dopamine D2/3 receptors. While our findings are consistent with findings from fMRI studies (e.g., Preuschoff et al., 2006) the temporal resolution of PET does not allow us to differentiate between anticipation and outcome evaluation in gambling. Furthermore, it is possible that other dopamine receptors, e.g., D1-class receptors, might interact with- and contribute to the dopamine dysfunctions in gambling disorder. Finally, the IGT performance in healthy controls was not reinforced by dopaminergic coding of uncertainty. The following sections therefore addresses the possible differences of dopamine functions in IGT performance between gambling disorder sufferers and healthy controls.

### Dopamine release and IGT performance in gambling disorder

To investigate adaptive learning functions of dopamine in IGT performance we (Linnet et al., 2011a) compared IGT performance in relation to dopamine release in the ventral striatum of 16 gambling disorder sufferers and 14 healthy controls. We used the regular ABCD version and the combined ABCD, KLMN and QRST versions, where group differences were measured as the average performance across the three different versions (ABCD + KLMN + QRST/3). The study compared overall group differences in IGT performance as well as group differences of IGT performance in relation to dopamine release in the ventral striatum.

A previous IGT study (Sevy et al., 2006) showed that pharmacological reduction of dopaminergic activity is associated with impaired IGT performance in healthy control volunteers, while increase of dopamine is associated with better IGT performance. We (Linnet et al., 2011a) therefore hypothesized that dopamine release in the ventral striatum would improve performance in healthy controls. Based on suggestions that risk and outcome uncertainty is associated with dopamine release in gambling disorder (Fiorillo et al., 2003), it was further hypothesized that dopamine release in the ventral striatum of gambling disorder sufferers would be associated with more risky decision-making, reflected in lower IGT performance.

**Figure 4:**
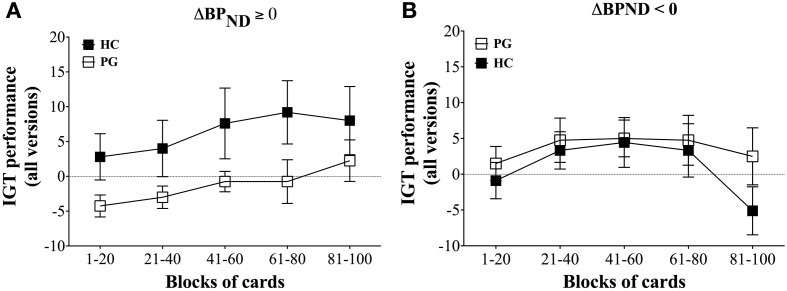
**IGT performance on ABCD version in relation to decrease (ΔBP_ND_ ≥ 0) and increase (ΔBP_ND_ < 0) in binding potential of ventral striatum. (A)** ΔBP_ND_ ≥ 0. Healthy controls (HC, black squares, *n* = 5) with binding potentials decrease (ΔBP_ND_ ≥ 0) in ventral striatum have significantly higher IGT performance on the ABCD version than gambling disorder sufferers (PG, white squares, *n* = 8), *F*_(5, 13)_ = 14.40, *p* < 0.0005. The abscissa shows trial blocks (1–20, 21–40, and so forth), while the ordinate shows the IGT performance across all versions. Mean and Standard Errors are illustrated in the squares and error bars, respectively. **(B)** ΔBP_ND_ < 0. Healthy controls (HC, black squares, *n* = 9) with increased binding potentials (ΔBP_ND_ ≥ 0) do not differ in IGT performance on the ABCD version compared with gambling disorder sufferers (PG, white squares, *n* = 8), *F*_(5, 17)_ = 1.78, ns. Reprint with permission from Linnet et al. (2011b).

The results showed that gambling disorder sufferers and healthy controls did not differ in IGT performance on the ABCD version or combined performance across the three tasks. However, when comparing IGT performance between gambling disorder sufferers and healthy controls dependent on dopamine release, a highly significant pattern emerged. Healthy controls with dopamine release in the ventral striatum had significantly higher IGT performance on the ABCD version than gambling disorder sufferers, *F*_(4, 11)_ = 14.40, *p* < 0.0005 (Figure 4A). In contrast, gambling disorder sufferers and healthy controls without dopamine release (dopamine inhibition) did not differ in IGT performance, *F*_(4, 15)_ = 1.78, *ns* (Figure 4B). Gambling disorder sufferers who released dopamine in the ventral striatum had significantly *lower* IGT performance than gambling disorder sufferers who did not release dopamine, *F*_(4, 14)_ = 8.25, *p* = 0.005, while healthy controls who released dopamine had significantly *higher* IGT performance than healthy controls who did not, *F*_(4, 12)_ = 4.85, *p* < 0.05.

The findings suggest that dopamine release was associated with adaptive behavior in healthy control individuals, but maladaptive behavior in gambling disorder sufferers. This might suggest that the function of dopamine differed between the two groups. Among gambling disorder sufferers the dopamine function appears to code uncertainty and reinforce risky and disadvantageous decision making. Among healthy controls the dopamine function appears to code outcome and reinforce adaptive and advantageous decision making. The dopamine dysfunctions and maladaptive gambling behavior in gambling disorder could further be fueled by the subjective experience of gambling. To address this aspect, the levels of gambling excitement were investigated.

#### Dopamine and subjective experience

Subjective gambling experiences such as increased excitement is central to gambling disorder (Neighbors et al., 2002; Rockloff and Dyer, 2006; Pantalon et al., 2008; Vachon and Bagby, 2009). Gambling excitement is associated with physiological measures of arousal (Moodie and Finnigan, 2005; Wulfert et al., 2005, 2008), and physiological arousal is generally increased during gambling (Leary and Dickerson, 1985; Dickerson et al., 1992; Coventry and Constable, 1999; Coventry and Hudson, 2001; Ladouceur et al., 2003; Moodie and Finnigan, 2005; Wulfert et al., 2005). Individuals with problem gambling or gambling disorder do not necessarily differ in physiological arousal from individuals without gambling problems (Griffiths, 1993; Sharpe et al., 1995; Coventry and Norman, 1997; Brown et al., 2004; Sodano and Wulfert, 2010), but some studies find an interaction between specific patterns of excitement and physiological arousal in gambling disorder (Goudriaan et al., 2006b). It is therefore, possible that a similar interaction exists between gambling excitement and dopaminergic neurotransmission in gambling disorder.

We (Linnet et al., 2011a) investigated the relation between subjective experience of gambling excitement and dopamine release in the ventral striatum of 18 gambling disorder sufferers and 16 healthy controls. It was hypothesized that dopamine release would be associated with increased excitement levels in gambling disorder sufferers compared with healthy controls.

Measures of excitement levels were obtained during PET scans, after each gambling round (ABCD, KLMN, and QRST). The computer automatically asked participants to rate their excitement level (“How exciting do you think the game is right now?”) on a scale ranging from 1 to 10, where 1 was the lowest rating and 10 was the highest.

**Figure 5:**
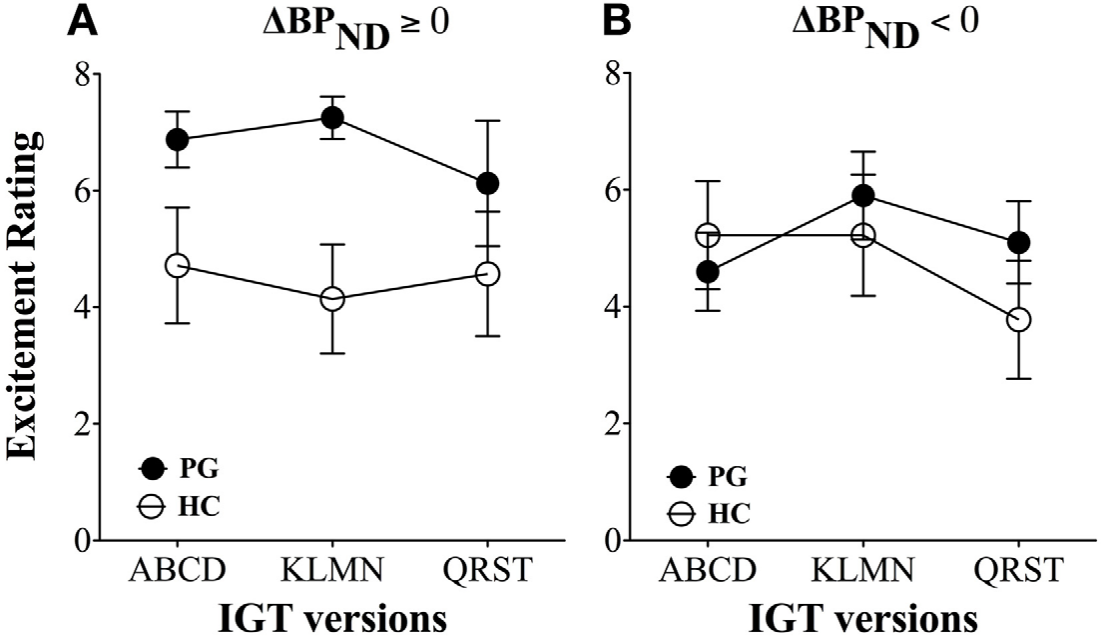
**Excitement levels between gambling disorder sufferers and healthy controls. (A)** Gambling disorder sufferers (PG, filled circles) with dopamine release (ΔBP_ND_ ≥ 0) have significantly higher excitement across games than healthy controls (HC, open circles) with dopamine release. **(B)** Gambling disorder sufferers (PG) and healthy controls (HC) without dopamine release (ΔBP_ND_ < 0) do not differ in excitement level across games. Reprint with permission from Linnet et al. (2011b).

The results showed that gambling disorder sufferers had significantly higher excitement levels than healthy controls throughout the three games, *F*_(2, 31)_ = 6.45, *p* = 0.01. However, these differences were due to increased excitement levels in gambling disorder sufferers with dopamine release. Gambling disorder sufferers with dopamine release had significantly higher excitement levels throughout the games than healthy controls with dopamine release, *F*_(2, 12)_ = 10.69, *p* < 0.005 (Figure 5A), while no differences in excitement levels were found between gambling disorder sufferers and healthy controls without dopamine release (dopamine inhibition) (Figure 5B). Gambling disorder sufferers with dopamine release also had significantly higher excitement levels than gambling disorder sufferers without dopamine release, *F*_(2, 15)_ = 6.94, *p* = 0.01, while there were no differences between healthy controls with dopamine release and healthy controls without dopamine release.

**Figure 6:**
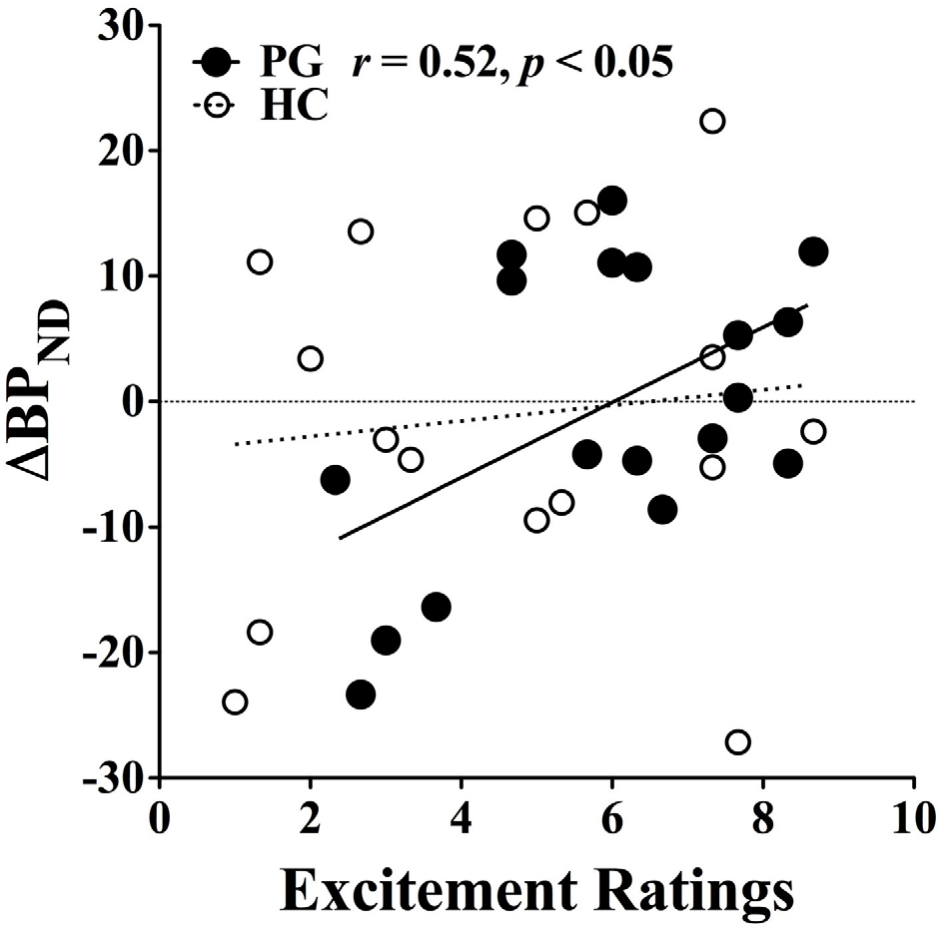
**Correlation between binding potential changes and excitement level.** Gambling disorder sufferers (PG, filled circles) show a significant correlation between excitement level on the abscissa and change in binding potential (ΔBP_ND_) on the ordinate, while the correlation fail to reach significance level in Healthy Controls (HC, open circles). Values above zero indicate dopamine release, while values below zero indicate dopamine inhibition. Reprint with permission from Linnet et al. (2011b).

Furthermore, there was a significant positive correlation between dopamine release and excitement level in gambling disorder sufferers, *r*_(18)_ = 0.52, *p* < 0.05, which did not reach significance level among healthy controls (see Figure 6). No linear interaction was found between excitement level and IGT performance or between IGT performance and dopamine release in either group. This suggests that the higher excitement levels in gambling disorder sufferers was specifically associated with increased dopamine release and not with better IGT performance.

These data might suggest that individuals with gambling disorder suffer from a dopaminergic “double deficit” condition, where dopamine release is associated with both impaired gambling behavior and increased excitement levels, and that both factors may contribute to the gambling disorder.

## Conclusion

The studies presented here point in the direction that gambling disorder sufferers: (1) do not have lower baseline dopamine binding; (2) do not have dopaminergic hypersensitivity toward gambling per se; (3) do not have dopaminergic hypersensitivity toward winning; (4) show dopaminergic sensitivity toward uncertainty in outcomes consistent with reward prediction error; (5) show maladaptive gambling behavior with dopamine release; and (6) show increased gambling excitement with dopamine release.

Together, the evidence suggests that dopamine is involved in adaptive as well as maladaptive decision making in gambling. Dopamine may guide and reinforce advantageous decision making, as seen in healthy controls, and may have helped these individuals develop a strategy and stay on task. From the perspective of reward prediction error, healthy controls might have taken a problem solving approach to the IGT, where the dopamine release was associated with a phasic dopamine response from the adaptive decision making of identifying advantageous decks. In other words, healthy controls received a dopaminergic “reward” from developing good strategies.

On the other hand dopamine might also be linked to disadvantageous decision making, and lead to long term losses, as seen in gambling disorder sufferers. From the perspective of dopaminergic coding of uncertainty, these individuals might have seen the IGT as a game of chance and assumed a more risk taking approach, where the dopamine release was associated with a sustained dopamine response from uncertainty. In other words, these individuals received a dopaminergic “reward” from uncertainty. Altogether, the dopaminergic dysfunctions may represent a “double deficit” condition, where dopaminergic dysfunctions toward risk and uncertainty reinforce maladaptive gambling behavior and increase excitement levels in gambling disorder.

However, the role of dopamine in gambling is complex and the suggestion of the three “fallacies” is therefore limited to the presented research. For instance, while there were no differences in PET measures of dopamine release between gambling disorder sufferers and healthy controls playing the IGT, there may be dopaminergic group differences in other contexts such as timing (e.g., dopaminergic activation in early or late anticipation or evaluation), type of game (e.g., real life gambling vs. IGT), motivational state etc.. For instance, the temporal resolution of PET does not allow differentiation between phasic and sustained dopamine response. Furthermore, the findings are limited to the level of dopamine D2/3 receptors; dopaminergic neurotransmission may differ at, e.g., the level of dopamine D1 receptors. Finally, the list is not exhaustive, i.e., there may be other types of “fallacies,” which challenge our understanding of the role of dopamine in gambling disorder and addiction.

In conclusion, the suggested “fallacies” and role of dopaminergic dysfunctions in the coding of reward prediction and uncertainty in gambling disorder presented here may serve as a starting point for further development of a dopaminergic model of addiction in gambling disorder and substance use disorders.

### Conflict of interest statement

The author declares that the research was conducted in the absence of any commercial or financial relationships that could be construed as a potential conflict of interest.
